# Embryos from Prepubertal Hyperglycemic Female Mice Respond Differentially to Oxygen Tension In Vitro

**DOI:** 10.3390/cells13110954

**Published:** 2024-05-30

**Authors:** Dhakshanya Predheepan, Sujith Raj Salian, Shubhashree Uppangala, Vani Lakshmi R, Guruprasad Kalthur, Borut Kovačič, Satish Kumar Adiga

**Affiliations:** 1 Centre of Excellence in Clinical Embryology, Department of Reproductive Science, Kasturba Medical College, Manipal Academy of Higher Education, Manipal 576104, India; dhakshanya.p@learner.manipal.edu (D.P.);; 2 Division of Reproductive Genetics, Department of Reproductive Science, Kasturba Medical College, Manipal Academy of Higher Education, Manipal 576104, India; shubha.u@manipal.edu; 3 Department of Data Science, Prasanna School of Public Health, Manipal Academy of Higher Education, Manipal 576104, India; vani.lakshmi@manipal.edu; 4 Division of Reproductive Biology, Department of Reproductive Science, Kasturba Medical College, Manipal Academy of Higher Education, Manipal 576104, India; guru.kalthur@manipal.edu; 5 Laboratory of Reproductive Biology, Department of Reproductive Medicine and Gynaecological Endocrinology, University Medical Centre, 2000 Maribor, Slovenia; borut.kovacic@ukc-mb.si

**Keywords:** oxygen tension, prepubertal hyperglycemia, embryo development, apoptosis

## Abstract

Reduced oxygen during embryo culture in human ART prevents embryo oxidative stress. Oxidative stress is also the major mechanism by which maternal diabetes impairs embryonic development. This study employed induced hyperglycemia prepubertal mice to mimic childhood diabetes to understand the effects of varying oxygen tension during in vitro embryonic development. The oocytes were fertilized and cultured at low (≈5%) oxygen (LOT) or atmospheric (≈20%) oxygen tension (HOT) for up to 96 h. Embryo development, apoptosis in blastocysts, inner cell mass (ICM) outgrowth proliferation, and *Hif1α* expression were assessed. Though the oocyte quality and meiotic spindle were not affected, the fertilization rate (94.86 ± 1.18 vs. 85.17 ± 2.81), blastocyst rate (80.92 ± 2.92 vs. 69.32 ± 2.54), and ICM proliferation ability (51.04 ± 9.22 vs. 17.08 ± 3.05) of the hyperglycemic embryos were significantly higher in the LOT compared to the HOT group. On the other hand, blastocysts from the hyperglycemic group, cultured at HOT, had a 1.5-fold increase in apoptotic cells compared to the control and lower *Hif1α* transcripts in ICM outgrowths compared to the LOT. Increased susceptibility of embryos from hyperglycemic mice to higher oxygen tension warrants the need to individualize the conditions for embryo culture systems in ART clinics, particularly when an endogenous maternal pathology affects the ovarian environment.

## 1. Introduction

Oxygen tension is one of the crucial factors for successful embryonic development in vitro [1]. Studies have reported that the oxygen (O_2_) tension in the mammalian intratubal and intrauterine environment is low, ranging from 2 to 8%, in contrast to atmospheric oxygen tension (≈20%) [2,3]. Low oxygen utilization during in vitro embryo culture may improve embryo viability, blastocyst rate, and hatching potential by increasing the activity of antioxidant enzymes and glucose transporter activities [4]. Similarly, human studies have reported the beneficial effects of low (5%) oxygen tension on blastocyst formation and fetal development [5]. Although the utilization of low O_2_ tension in Assisted Reproductive Technology (ART) cycles has resulted in better embryo quality and pregnancy rates [2], a meta-analysis has indicated that the available evidence supporting the beneficial effects of low oxygen is of poor quality and inconclusive [6].

Studies have shown that follicle development and ovarian function are both affected by chronic hyperglycemia [7,8], which further increases the risk of infertility [9,10]. Due to the prevalence of childhood diabetes, early onset of reproductive abnormalities is also possible [10]. A recent study has shown that chronic hyperglycemia in the prepubescent period negatively impacts mouse oocyte functional competence and spindle integrity during the reproductive period [11]. Additionally, hyperglycemia is known to be associated with decreased fertility and impaired embryonic development [7]. The reactive oxygen species (ROS) levels of embryos developed under high oxygen tension were greater than those of embryos developed under low oxygen [12]. Eventually, the oxidative stress induced by ROS can affect the embryonic epigenome [12,13], disrupt transcriptional regulation, and impair embryonic development [14]. Oxidative stress is a major mechanism by which diabetes may impair embryonic development [15]. Importantly, ART laboratories do not follow individualized protocols for patient-specific health conditions due to a lack of scientific research. Therefore, understanding the behavior of embryos resulting from the oocytes of diabetic mice and their susceptibility to varying oxygen tension can provide valuable information to ART laboratories. We hypothesized that embryos derived from oocytes exposed to prolonged hyperglycemia might have a differential response to varying oxygen tension. To address this, hyperglycemia was induced chemically in prepubertal mice, and upon puberty, the superovulated oocytes were assessed for meiotic integrity, fertilizing ability, and developmental competence in both physiological (5%) and atmospheric (20%) O_2_ environments. Fertilization rate, embryo development and quality, blastocyst development rate, apoptosis, and inner cell mass (ICM) proliferation were evaluated and compared between the two oxygen environments.

## 2. Materials and Methods

### 2.1. Animals and Ethical Clearance

Swiss albino mice were housed in the Central Animal Research Facility of the Manipal Academy of Higher Education. Six mice per cage were housed under controlled conditions of 23 ± 2 °C, a 12 h light–dark cycle, and 50 ± 5% humidity, and fed with a standard diet and water ad libitum. Animal handling and research experiments were conducted as per the guidelines of the Institutional Animal Ethics Committee (IAEC/KMC/11/2021 and IAEC/KMC/18/2021). Animals were allowed to acclimatize to the facility for 2 weeks before being put on trial/experiment. A minimum of three trials were performed for each outcome measure to ensure the reproducibility of the results.

### 2.2. Experimental Design

Initially, the blood was withdrawn from the tail vein of the 3-week-old female mice, and the initial blood glucose readings were acquired using a glucometer (Accu-Chek^®^ Active, Roche Diagnostics GmbH, Mannheim, Germany) to confirm the baseline blood glucose level prior to STZ injections. As previously reported [16], three successive doses of 75 mg/kg body weight of streptozotocin (STZ) (Cat. No.: S0130, Sigma Aldrich, St. Louis, MO, USA) were administered intraperitoneally to 3-week-old female Swiss albino mice (N = 37). The vehicle control (VC) (N = 24) received an equal amount of 0.01 M sodium citrate buffer. The control (C) group (N = 20) received no injections. From the third day after the first injection of STZ, the blood glucose level was checked once every 3 days, and mice with blood glucose levels > 250 mg/dL were considered hyperglycemic. All investigations were performed three weeks following their last injection or during their sixth week of life. All of the analysis was performed manually by two independent investigators. Slides/study groups were coded to avoid observer bias.

### 2.3. Ovarian Stimulation

Female mice were superovulated by injecting 5 IU of pregnant mare serum gonadotropin (PMSG, Sigma Aldrich, St. Louis, MO, USA) intraperitoneally, followed by 10 IU of human chorionic gonadotropin (hCG, Eutrig-HP) 48 h later. Animals were weighed and sacrificed 13 h post-hCG injection to retrieve the oocyte cumulus complex (OCC).

### 2.4. Assessment of Spindle Morphology

OCCs were exposed to hyaluronidase (Cat. No.: H4272, Sigma Aldrich, St. Louis, MO, USA), and then, denuded oocytes were assessed for maturity, morphology, and meiotic spindle structure. Metaphase II oocytes were rinsed in PBS supplemented with 0.1% BSA and permeabilized at 37 °C for 1 h using extraction buffer (50 mM potassium chloride, Cat. No: P5405, Sigma Aldrich, St. Louis, MO, USA; 5 mM ethylenediaminetetraacetic acid disodium salt, Cat. No.: 54960, Sisco Research Laboratories, Maharashtra, India; 0.5 mM magnesium chloride, Cat. No: 1349130, Sisco Research Laboratories, Maharashtra, India; 25 mM HEPES, Cat. No.: H-3375, Sigma Aldrich, St. Louis, MO, USA; 25% glycerol, Cat. No: G9012, Sigma Aldrich, St. Louis, MO, USA; 2% Triton X-100, Cat. No.: 1.8603.1000, Merck; 20 μM phenylmethane sulphonyl fluoride, Cat. No: 1592, Himedia, Maharashtra, India) adjusted to pH 6.75. Oocytes were then fixed using ice-cold methanol for 15 min at −20 °C, followed by blocking using 5% knockout serum (Cat. No.: 10828-010, Gibco, Billings, MT, USA) and 0.25% Triton-X for 1 h at 37 °C. The oocytes were incubated overnight at 4 °C in 1:150 diluted primary anti-α-tubulin antibody (Cat. No.: T9026; RRID: AB_477593; Sigma Aldrich, St. Louis, MO, USA) followed by treatment with 1:500 FITC-tagged goat anti-mouse IgG antibody (Cat. No.: NB7538; RRID: AB_524787; Novus Biologicals, Centennial, CO, USA) for 1 h at 37 °C. The chromosomes were stained with 4 µg/mL 4′,6-Diamidino-2-phenylindole (Cat. No.: D9542, Sigma Aldrich, USA) and observed under a fluorescent microscope (Imager-A1, Zeiss, Gottingen, Germany).

### 2.5. In Vitro Fertilization and Preimplantation Embryo Development

Cauda epididymis from 10–12-week-old Swiss albino male mice were retrieved in pre-warmed Earl’s balanced salt solution (EBSS) (Cat. No.: E2888, Sigma Aldrich, St. Louis, MO, USA) supplemented with 0.1% bovine serum albumin (BSA) (Cat. No.: A3311, Sigma Aldrich, St. Louis, MO, USA) and teased to release spermatozoa. The sperm suspension was centrifuged, and the pellet was overlaid with Potassium Simplex Optimization Medium (KSOM-AA, Cat. No.: MR-107-D, Sigma Aldrich, St. Louis, MO, USA) supplemented with 0.1% BSA and incubated for swim-up for 45 min at 37 °C. The supernatant containing the motile spermatozoa was then collected, inseminated in a 4-well dish (Cat. No.: 176740, Thermo Fisher Scientific, Waltham, MA, USA), and overlaid with oil. Then, both of the oviducts from Swiss albino female mice were collected, and each oviduct was transferred to separate collection tubes with EBSS supplemented with 0.1% BSA. The oviduct collected was gently cut open to release the OCC into EBSS containing 0.1% BSA. OCCs were transferred to the 4-well dish and co-incubated with washed spermatozoa (2 × 10^6^ spermatozoa/mL). OCCs from one oviduct were transferred to 5% O_2_ or low oxygen tension (hereafter referred to as LOT) and 20% O_2_ or high oxygen tension (hereafter referred to as HOT). The gametes were co-incubated at 37 °C with 5% CO_2_ at two different oxygen tensions (LOT and HOT) and assessed for fertilization at 10.5 h post-insemination (hpi) to understand the fertilizing ability in varying oxygen environments. The oocytes were then washed in KSOM supplemented with 0.1% BSA, and they were either categorized as normally fertilized (containing two pronuclei and two polar bodies) or abnormally fertilized (with one or no pronuclei). Only normally fertilized oocytes (5 oocytes in 20 µL droplet) were transferred to fresh KSOM supplemented with 0.1% BSA and cultured at 37 °C with 5% CO_2_ at LOT and HOT conditions until 96 hpi. Embryos were assessed using an inverted microscope (IX73, Olympus, Tokyo, Japan) every 24 hpi (24, 48, 72, 96, and 108 hpi) to determine their developmental competence at both LOT and HOT.

### 2.6. TUNEL Assay

Apoptosis in blastocysts was assessed by terminal deoxynucleotidyl transferase dUTP nick end labeling (TUNEL) assay (In Situ Cell Death Detection Kit, TMR red, Cat. No.: 12156792910; Roche, Mannheim, Germany) because DNA fragmentation in apoptosis is predominantly associated with embryo quality. The In Situ Cell Death Detection Kit, TMR red, is based on the detection of single- and double-stranded DNA breaks that occur at the early stages of apoptosis. At 96 hpi, the blastocysts were rinsed thrice in phosphate-buffered saline (PBS), transferred to a multi-well plate (Cat. No.: 163118, Thermo Fisher Scientific, Waltham, MA, USA), and fixed overnight with 4% paraformaldehyde (*w*/*v*). The embryos were then rinsed three times in PBS, followed by one-hour permeabilization in permeabilization buffer (0.1% sodium citrate (*w*/*v*), 0.5% BSA (*w*/*v*), and 0.5% Triton X-100 (*v*/*v*) in PBS) at room temperature. The embryos were washed thrice in PBS containing 0.5% BSA and treated for 1 h at 37 °C with the TUNEL reaction mixture (9:1 ratio of TMR red labeling solution to enzyme solution). During the incubation period, the enzyme terminal deoxynucleotidyl transferase (TdT) facilitates the addition of TMR-dUTP at the free 3′-OH groups in both single-stranded and double-stranded DNA breaks. The embryos were washed and then counterstained with 4 µg/mL DAPI (4′,6′-diamino-2-phenylindole) to stain the nucleus, thereby assessing the total cell number. Embryos were then transferred to clean microscopic slides and examined under a fluorescence microscope (Carl Zeiss, Gottingen, Germany) to determine the total cell number and TUNEL-positive cells. The labeling index was calculated as the percentage of TUNEL-positive cells per blastocyst.

### 2.7. ICM Outgrowth Assay

The ability of the blastocyst to attach and proliferate post-implantation in vitro was assessed using the ICM outgrowth assay because it is crucial to comprehend the in vitro proliferation ability beyond the blastocyst stage. So, the blastocysts appearing morphologically normal with expanded blastocoeles at 108 hpi were selected for ICM outgrowth assay, which was performed as described earlier [17], with minor modifications. Briefly, multi-well dishes (Cat. No.: 07-200-92, Thermo Fisher Scientific, Waltham, MA, USA) were precoated with 0.1% gelatin (Cat. No.: G1393, Sigma-Aldrich, St. Louis, MO, USA) for 30 min at room temperature. Excess gelatin was removed, and the dishes were air-dried. Individual blastocysts at 108 hpi were transferred into each well containing 200 µL DMEM (Cat. No.: D5648, Sigma-Aldrich, St. Louis, MO, USA) supplemented with 20% fetal bovine serum (FBS, Cat. No.: 10270106, Gibco, Billings, MT, USA), overlaid with oil and cultured until 204 hpi under LOT and HOT environments. Blastocyst attachment and proliferation were monitored at 156 hpi under an inverted phase contrast microscope (IX 73, Olympus, Japan). The ICM outgrowths (IO) at 204 hpi were classified based on the morphology and size of the outgrowths, as reported previously [17]: completely developed ICM outgrowth (CIO), large ICM outgrowth (LIO), small ICM outgrowth (SIO), and no ICM outgrowth (NIO). The images were captured using Olympus IX 73, Olympus, Tokyo, Japan.

### 2.8. Isolation of Total RNA, cDNA Synthesis, and Gene Expression Analysis

Total RNA was extracted from a minimum of 10 ICM outgrowths at 204 hpi (CIO and LIO) as per the kit recommendations (15596018, Ambion Life Technologies, Austin, TX, USA). Following the manufacturer’s instructions, 53 ng of total RNA was reverse-transcribed using random primers and a high-capacity cDNA RT kit (4368814, Applied Biosystems, San Francisco, CA, USA). Using the SYBR green kit (Cat. No.: RR420A, DSS TAKARA BIO INDIA Pvt. Ltd., Karnataka, India) and the StepOneTM Real-Time PCR System, a quantitative polymerase chain reaction (qPCR) was performed (Thermo Fisher Scientific, Waltham, MA, USA). The SYBR green assay (Sigma Aldrich, St. Louis, MO, USA) for *Hif1α* (5′ CCTGCACTGAATCAAGAGGTTGC and 3′ CCATCAGAAGGACTTGCTGGCT) was used. The qPCR outcomes were standardized using actin (5′ CATTGCTGACAGGATGCAGAAGG and 3′ TGCTGGAAGGTGGACAGTGAGG) as a reference gene. Due to the proximity to physiological conditions, ICM outgrowths of control female mice cultured at LOT were used as a reference sample for studying *Hif1α* expression in ICM outgrowths. The qPCR results were analyzed by the delta–delta Ct method.

### 2.9. Statistical Analysis

The data are represented as mean ± standard error of the mean (SEM). Based on the distribution of the data points, either the one-way Analysis of Variance (ANOVA) or the Kruskal–Wallis test was used to analyze the data. The unpaired *t*-test or Mann–Whitney U test was used to analyze the data between the groups. The data for assessing spindle abnormality were evaluated by using the chi-square test. All statistical tests were conducted using the GraphPad Instat software version 3.0 (Graphpad Inc., La Jolla, CA, USA). GraphPad Prism 8 (GraphPad Prism software, La Jolla, CA, USA) was used to provide the graphical representation of the data. The level of significance was set at 5% throughout the study. All of the data provided are from a minimum of three independent trials.

## 3. Results

### 3.1. Fertilization, Developmental Competence, and Meiotic Integrity of Hyperglycemic Oocytes

**Figure 1:**
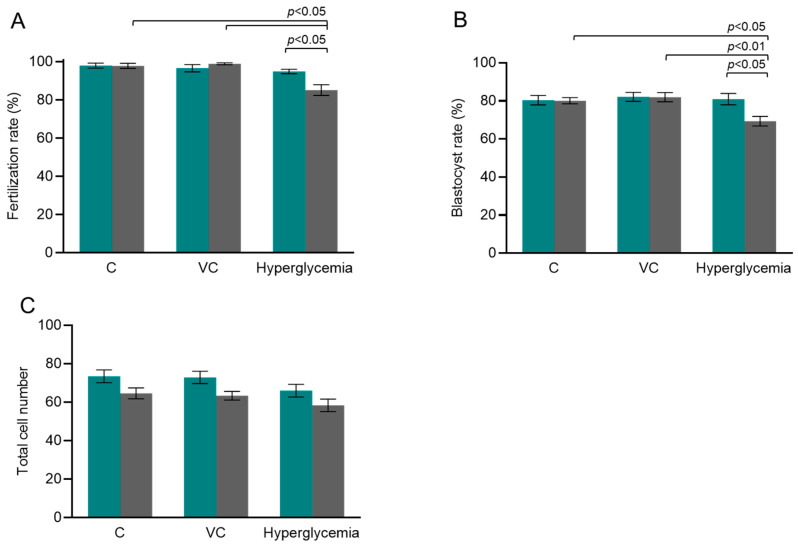
The influence of oxygen tension on in vitro fertilization and embryo development of oocytes. (**A**) Fertilization rate (10 hpi) at LOT (teal bar): N = 181, 203, and 308 for the control (C), vehicle control (VC), and hyperglycemic groups, respectively; at HOT (gray bar): N = 155, 248, and 298 for the C, VC, and hyperglycemic groups, respectively, where ‘N’ indicates the number of metaphase II oocytes subjected to fertilization. (**B**) Blastocyst rate (96 hpi) at LOT (teal bar): N = 176, 195, 293 for the C, VC, and hyperglycemic groups, respectively; at HOT (gray bar): N = 150, 245, and 257 for the C, VC, and hyperglycemic groups, respectively, where ‘N’ indicates the number of fertilized oocytes. (**C**) Total cell number of blastocysts at LOT (teal bar): N = 56, 41, and 44 for the C, VC, and hyperglycemic groups, respectively; at HOT (gray bar): N = 47, 51, and 38 for the C, VC, and hyperglycemic groups, respectively. ‘N’ indicates the number of blastocysts included in the analysis. The number of female Swiss albino mice used for 5 independent IVF trials of the C, VC, and hyperglycemic groups are 17, 22, and 34, respectively. One-way ANOVA was used to analyze the data on oocyte number per animal and maturation rate between the C, VC, and hyperglycemic groups. The Kruskal–Wallis test was used to analyze the data between the C, VC, and hyperglycemic groups for fertilization rate and total cell number, whereas one-way ANOVA was used to analyze the data between the C, VC, and hyperglycemic groups for blastocyst rate. Further, an unpaired *t*-test was used to analyze the data between the sub-groups (LOT and HOT) for fertilization rate and blastocyst rate, and the Mann–Whitney U test was used when total cell number was evaluated. The chi-square test was used to assess the spindle abnormality.

**Figure 2:**
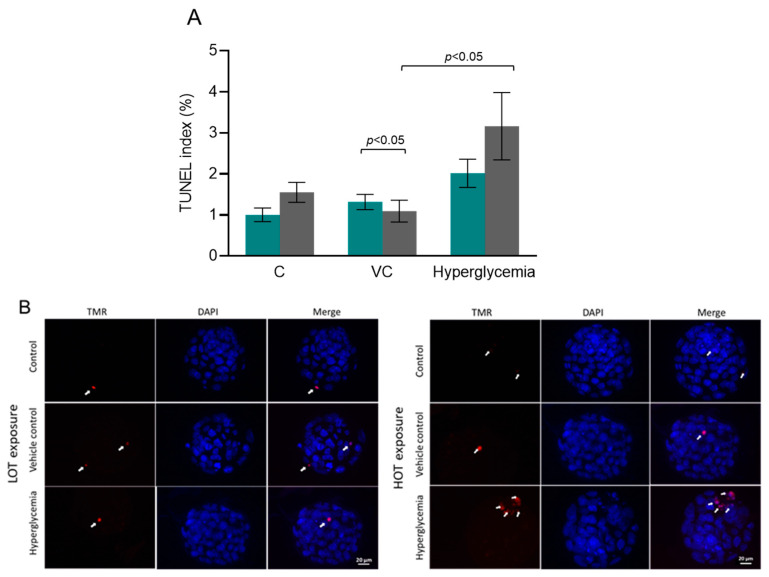
The effect of oxygen tension on apoptosis in blastocysts. (**A**) TUNEL index at LOT (teal bar): N = 56, 41, and 44 for the C, VC, and hyperglycemic groups, respectively; at HOT (gray bar): N = 47, 51, and 38 for the C, VC, and hyperglycemic groups, respectively. (**B**) Representative images of TUNEL assay in blastocysts (40× (scale bar: 20 µm). ‘N’ indicates the number of blastocysts included in the analysis. The white arrows indicate the TUNEL-positive cells. The Kruskal–Wallis test was used to analyze the data between the C, VC, and hyperglycemic groups, whereas the Mann–Whitney U test was used to analyze the data between the sub-groups (LOT and HOT) when the apoptotic index was evaluated.

The oocytes collected from the superovulated animals were assessed for nuclear maturity, morphology, and meiotic spindle structure prior to exposing them to varying oxygen tension in vitro. When oocyte quality was compared between in vivo matured control and hyperglycemic groups, a moderate decline in the oocyte number per animal ((21.82 ± 1.15, N = 17) vs. (20.74 ± 1.67, N = 34)), a decline in the oocyte maturation rate ((91.46 ± 2.45, N = 17) vs. (85.29 ± 6.56, N = 34)), and meiotic spindle abnormality of the oocytes ((10.91%, *n* = 55) vs. (13.85%, *n* = 65)) were observed. However, the differences were not statistically significant. On the other hand, when IVF was attempted at varying oxygen tensions, the co-incubation of gametes resulted in a significantly lower fertilization rate (85.17 ± 2.81, N = 298) (*p* < 0.05) of oocytes in the HOT group when compared to the LOT group (94.86 ± 1.18, N = 308) (Figure 1A) within the hyperglycemic group. Further, the prepubertal hyperglycemic oocytes exposed to HOT (85.17 ± 2.81, N = 298) had significantly (*p* < 0.05) lower fertilizing ability when compared to the C (97.87 ± 1.31, N = 155) and VC (98.93 ± 0.48, N = 248) groups. Moreover, the prepubertal hyperglycemic oocytes exposed to HOT had significantly lower (*p* < 0.05) blastocyst-forming potential in the HOT group (69.32 ± 2.54, N = 257) when compared to the LOT group (80.92 ± 2.92, N = 293). Furthermore, the prepubertal hyperglycemic oocytes exposed to HOT had significantly (*p* < 0.05–0.01) lower blastocyst-forming potential when compared to the C (80.07 ± 1.60, N = 150) and VC (81.97 ± 2.43, N = 245) groups (Figure 1B). However, the total cell number was comparable between the LOT and HOT groups within the C, VC, and hyperglycemic groups at 96 hpi (Figure 1C and Figure 2B).

### 3.2. Increased Incidence of Apoptosis in Blastocysts from HOT Culture

The TUNEL labeling was performed to investigate apoptosis in blastocysts. Importantly, the number of apoptotic cells in hyperglycemic embryos in the HOT group were approximately 1.5-fold higher than in the LOT group, though differences were not statistically significant according to the Mann–Whitney test (Figure 2A). However, a significant difference was observed between the HOT group and the corresponding vehicle control group (*p* < 0.05). The representative images of the total cell number and the apoptotic cells of the embryos are shown in Figure 2B.

### 3.3. HOT Impaired ICM Expansion and Hif1α Expression at 96 h of Extended In Vitro Culture

**Figure 3:**
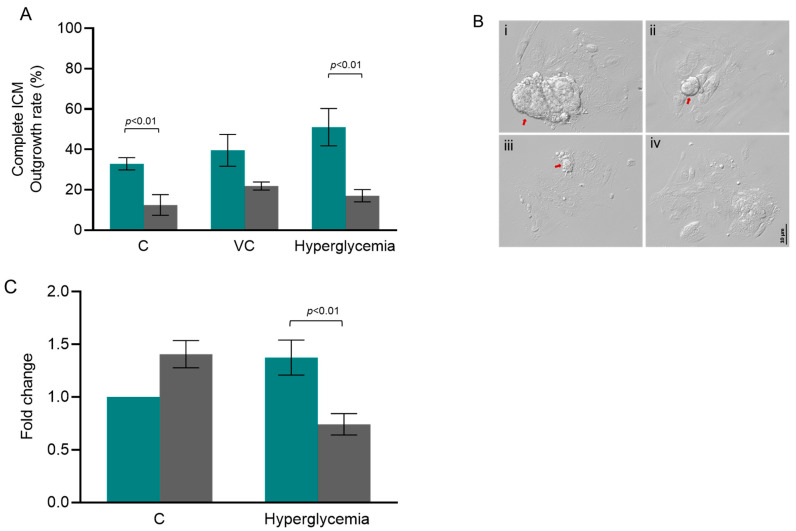
The influence of oxygen tension on ICM outgrowth. (**A**) Complete ICM outgrowth rate at LOT (teal bar): N = 83, 64, and 61 for the C, VC, and hyperglycemic groups, respectively; at HOT (gray bar): N = 79, 96, and 94 for the C, VC, and hyperglycemic groups, respectively. ‘N’ indicates the number of blastocysts included in the analysis from 4 independent trials. (**B**) Representative phase-contrast images of ICM outgrowths at 204 hpi: (i) completely developed ICM outgrowth (CIO), (ii) large ICM outgrowth (LIO), (iii) small ICM outgrowth (SIO), and (iv) no ICM outgrowth (NIO), respectively (40×). The red arrows indicate the ICM outgrowths (scale bar: 10 μm). (**C**) The effect of varying oxygen tension on the mRNA level of *Hif1α* in ICM outgrowths of embryos from the hyperglycemic group (N = 4 trials). ICM outgrowths of control (C) female mice cultured at LOT were used as the reference sample due to their proximity to physiological conditions. *Actb* served as a reference gene. The Kruskal–Wallis test was used to analyze the data between the C, VC, and hyperglycemic groups, whereas the Mann–Whitney U test was used to analyze the data between the LOT and HOT groups when the complete ICM outgrowth rate was evaluated. One-way ANOVA was used to analyze the statistics of *Hif1α* expression.

ICM outgrowth rate after 96 h of extended culture was significantly reduced in the HOT group when compared to the LOT group (*p* < 0.05) (Figure 3A) within both the control and hyperglycemic groups. Additionally, the mRNA transcripts of *Hif1α* showed about a significant (*p* < 0.01) 2-fold reduction in ICM outgrowths of hyperglycemic embryos from the HOT group when compared to the hyperglycemic embryos from the LOT group, but not significant when compared to the control embryos from the LOT group (reference sample control) (Figure 3C). On the other hand, *Hif1α* expression was not significantly different in ICM outgrowths of control embryos cultured in LOT and HOT conditions.

## 4. Discussion

This is the first approach to elucidate the influence of oxygen tension on the preimplantation embryos derived from oocytes exposed to experimentally induced hyperglycemia during prepubertal life in mice. The key findings of this study revealed that HOT impairs pre- and peri-implantation embryo development in vitro as indicated by the reduced fertilizing ability and blastocyst-forming potential, increased apoptosis in blastocysts, reduced ICM proliferation, and deregulation of *Hif1α* transcripts in ICM outgrowths of hyperglycemic embryos.

Embryo culture in LOT has become a standard of good practice in in vitro fertilization (IVF) laboratories [18,19], though clinical outcomes from this approach are still contradictory. In many laboratories across the globe, embryo culture is still performed in atmospheric (20%) O_2_ concentrations, whereas the physiological intrauterine O_2_ concentration is between 2 and 6% [20]. Reducing the O_2_ tension requires using nitrogen gas to purge O_2_ out of the incubator, and the process requires special sensors and equipment, which add costs and financial burden to the center. Earlier studies have suggested that high O_2_ concentration during in vitro culture can increase oxidative stress and lead to the developmental blockage of embryos [21,22]. These observations raise concerns about the susceptibility of embryos to HOT, especially when there is a maternal pathology.

Acute prepubertal hyperglycemia is known to delay meiotic progression and increase spindle defects in oocytes [23]. On the other hand, when prepubertal mouse ovaries were exposed to prolonged hyperglycemia, germinal vesicle (GV) stage oocytes experienced a significant reduction in the germinal vesicle breakdown (GVBD) process, an increased frequency of meiotic spindle defects, and chromosome misalignment after in vitro maturation (IVM) [15]. In the present study, surprisingly, when ovulated oocytes from hyperglycemic mice were assessed for maturity and spindle structure, only a moderate but non-significant increase in the spindle defects was observed. Since in vitro matured oocytes have an increased susceptibility to experience structural and functional defects compared to in vivo matured oocytes [24], it is possible that hyperglycemia-induced endogenous stress could have sensitized the spindle structure to lose its integrity during the process of IVM. However, further research is required to elucidate the mechanism behind this interesting observation.

When fertilization was attempted in ovulated oocytes under varying oxygen tension conditions, HOT significantly reduced the fertilizing ability of hyperglycemic oocytes compared to LOT, which could be attributed to a high-oxygen environment. Impairment in embryonic development was observed when a hyperglycemic state was induced in experimental animals [25]. However, in the present study, the blastocyst-forming potential of the hyperglycemic embryos was significantly reduced when embryos were cultured at HOT compared to LOT. Importantly, apoptosis during in vitro embryo culture is likely a result of less-than-ideal conditions and may therefore serve as an indicator of the embryos’ quality [26]. Studies have reported that hyperglycemia induces apoptosis in preimplantation embryos [27,28]. Notably, few studies have revealed that in a controlled physiological process, cells produce ROS [29]. Yet, an increase in ROS is harmful and causes oxidative stress, leading to increased apoptosis [30]. Though this study did not conduct specific experiments to measure ROS levels, our findings were consistent with these reports, revealing that the apoptosis in hyperglycemic embryos cultured at HOT was considerably higher than in control embryos and indicating that the exposure of embryos derived from diabetic mice to HOT may be detrimental. Nevertheless, it is imperative to conduct additional research on the upstream regulators of apoptosis to gain a more comprehensive understanding of the relationship between oxidative stress and apoptosis. Further, it is important to note that the significant difference observed between the HOT and LOT in TUNEL assay results of the vehicle control groups can be attributed to the fact that 50% of the TUNEL index values in the HOT group were zeros (0), resulting in a median value of zero (0). However, this finding is inconsequential and lacks scientific significance.

Genetically abnormal embryos could progress until the blastocyst stage due to limited checkpoint response during preimplantation development [31]. Hence, assessing the developmental competence till the blastocyst stage alone is not a reliable indicator of implantation potential. Human studies suggest that the exposure of cells to an atmospheric (20%) oxygen system may not be restricted to embryos alone but may also directly affect the self-renewal of human embryonic stem cells, which is dependent on their ability to regulate their energy metabolism [32]. ICM outgrowth culture provides a more sensitive screening method for IVF quality control systems [33] and hence can be used as a surrogate marker for embryo implantation potential. The findings of our study indicate that exposure to HOT has a negative impact on the ability of the ICM to proliferate. This is supported by the observed inability of the ICM to develop into complete outgrowths, as confirmed through morphological analysis. Importantly, hyperglycemic embryos cultured at HOT had significantly declined ICM outgrowth formation, indicating the detrimental effect beyond the blastocyst stage. Nevertheless, this assertion lacks further substantiation as pluripotent markers were not employed to stain and distinguish the ICM and trophectoderm (TE) cells.

The transcription factor hypoxia-inducible factor 1 alpha (*Hif1α*) is expressed in both male and female germlines and is essential for forming early progenitor cells [34]. The *Hif1α* gene protects embryos from intrauterine hypoxia and promotes angiogenesis during embryo development [35]. Importantly, *Hif1α* is also required for the normal development of the placenta and for fetal heart development from embryonic day 7.75 to 15, and a loss of *Hif1α* is likely to result in defective myocardium development and embryo lethality by embryonic day 10.5 [36]. HIF-1 protein translocation from the cytoplasm to the nucleus was found to be higher in the low (5%)-O_2_ and dynamic shift (5% to 2%) O_2_ groups than in the high (20%)-O_2_ group [37], suggesting that the expression of *Hif1* in a low-oxygen environment may benefit the preimplantation mouse embryos. In accordance with these findings, our observation revealed a significant downregulation of *Hif1α* transcripts in ICM outgrowths of hyperglycemic embryos cultured under HOT, unlike in the ICM outgrowths of the control (C) embryos, indicating that the deregulation of *Hif1α* in hyperglycemic conditions might be specific to oxygen tension.

Moreover, it is vital to examine the facet in which scientific studies have demonstrated the influence of potential antioxidants employed to reduce ROS during in vitro embryo development. Antioxidants function as agents that scavenge free radicals, provide protection to cells, and facilitate the repair of any damage caused by free radicals. Furthermore, numerous studies have demonstrated that the incorporation of antioxidants into the culture medium enhances in vitro embryo development [38,39]. The strength of this study is the use of a prepubertal mouse model to assess the influence of hyperglycemia on fertilization and embryo developmental potential in relation to oxygen tension. We believe that the model used in our setup can mimic childhood diabetes. The mouse model provides reliable genetic and physiological conditions for the experimental approach in situations that cannot be completely mimicked in humans due to the technical complexity of designing the study. In a clinical scenario, children and young adults diagnosed with diabetes may not experience prolonged hyperglycemia if treated effectively. Consequently, this model is constrained by the absence of a cohort of hyperglycemic mice receiving treatment. Nonetheless, delayed/a lack of diagnosis or improper treatment follow-up may lead to chronic hyperglycemic exposure in diabetic children. When such individuals seek ART, laboratories should consider additional precautions and optimize the culture conditions to reduce the risk to embryos.

## 5. Conclusions

The preliminary findings from this study suggest that embryos developed from oocytes exposed to prepubertal hyperglycemia have increased susceptibility to higher oxygen tension during in vitro culture. This observation warrants the need to individualize the conditions for embryo culture systems in ART clinics, especially when an endogenous maternal pathology affects the ovary.

## Data Availability

The original data presented in this study are openly available in the open science framework at https://osf.io/xudj2/files/osfstorage/663f91244664da6ad3ed6ac8, accessed on 11 May 2024.
